# Long-Term Effects of Neonatal Single or Multiple Isoflurane Exposures on Spatial Memory in Rats

**DOI:** 10.3389/fneur.2013.00087

**Published:** 2013-07-08

**Authors:** Kathy L. Murphy, Mark G. Baxter

**Affiliations:** ^1^Department of Experimental Psychology, University of Oxford, Oxford, UK; ^2^Glickenhaus Laboratory of Neuropsychology, Department of Neuroscience, Friedman Brain Institute, Icahn School of Medicine at Mount Sinai, New York, NY, USA

**Keywords:** pediatric anesthesia, anesthetic neurotoxicity, neurodevelopment, cognitive impairment, Hippocampus

## Abstract

General anesthetics are neurotoxic to neonatal rodents and non-human primates. Neonatal exposure to general anesthetics has been associated with long-term cognitive deficits in animal models. Some data from humans are consistent with long-term deleterious effects of anesthetic exposure early in life on cognitive development, with multiple exposures to general anesthetics being particularly damaging. We sought to determine whether repeated exposure of neonatal rats to anesthesia was associated with long-term cognitive impairments and whether the magnitude of impairments was greater than that resulting from a single exposure. Male or female Long–Evans rat pups were exposed to 1.8% isoflurane for 2 h on postnatal day (P) 7, or for 2 h each on P7, P10, and P13. Testing in a spatial working memory task began on P91. Rats that were repeatedly exposed to isoflurane were impaired relative to controls in the spatial working memory task. Male rats that received a single exposure to isoflurane showed an unexpected facilitation in spatial memory performance. These results support the hypothesis that multiple neonatal exposures to general anesthesia are associated with greater long-term cognitive impairment than a single exposure. The findings are congruent with human epidemiological studies reporting long-term cognitive impairments following multiple but not single general anesthetics early in life.

## Introduction

Exposure to anesthetic agents early in development can cause neurotoxicity and neonatal exposure in rodents is associated with persistent deficits in cognition in later adulthood, particularly in learning and memory (1, 2). These deficits have been demonstrated following exposure to a variety of anesthetic agents (3–5) and are not restricted to agents of a particular drug class. Deficits occur following exposure at different perinatal developmental timepoints (4, 6–9) although the most commonly demonstrated effect in rodents occurs following exposure at postnatal day (P) 7, a critical period of synaptic growth in brain development. Anesthetic-induced cognitive deficits persist into adulthood, are thought to be dose and duration dependent, and some evidence suggests that they are progressive (9, 10).

The mechanism by which cognitive impairment occurs is currently unclear (11). Exposure to anesthesia causes widespread neuroapoptosis in the developing brain of a variety of species (12–16) and the greatest vulnerability appears to occur at the peak of synaptogenesis (15, 16) implying that neuronal loss early in development leads to cognitive impairments later in life. There are, however, likely to be other mechanisms by which anesthesia causes neural dysfunction and cognitive impairment, including disturbances in synaptic morphology in surviving neurons (for review 17).

Retrospective human patient studies have shown that children who receive multiple but not single anesthetics at a young age have an increased risk of learning disability (18, 19) and attention deficit disorder (20). This raises the question: are multiple exposures to anesthesia worse than a single exposure in terms of long-term cognitive outcome? Of course, retrospective patient studies cannot distinguish whether anesthesia *per se*, or the need for anesthesia, leads to this increased risk. Children that require anesthesia may also experience a range of conditions that could influence their cognitive development, such as surgical procedures, co-morbidities, and missed schooling. Prospective multicentre studies are clearly needed to begin to understand the effect these variables have on cognitive outcome, and such studies are now underway (21). Patient studies cannot, however, exclude such variables when investigating the question of relative effects of single and multiple exposures to anesthesia. In order to achieve this an *in vivo* animal model is required.

Multiple exposure of neonatal mice to sevoflurane has recently been shown to result in cognitive deficits, when testing is conducted 1 month post exposure. Perhaps unexpectedly, the same study demonstated that the cognitive performance of single-exposed mice was enhanced (22). This study also reported that there were no effects of desflurane on cognitive performance, indicating that anesthetic induced cognitive impairment is agent selective. Multiple exposure of neonatal rodents to isoflurane (a commonly used volatile anesthetic agent and the one chosen for this study) has been shown to cause long-term cognitive deficits, but the effects of multiple exposures were not compared to those of a single exposure (9). The extent to which cross-study comparisons can be made is limited, as the conduct of behavioral experiments is inherently variable and disparity exists regarding whether a single dose of anesthesia is, or is not (22–24) sufficient to cause long-term cognitive impairment. The goal of the current study, therefore, was to directly compare the performance of adult rats, exposed to either single or multiple episodes of isoflurane anesthesia as neonates, on the acquisition of a spatial memory task. This addresses the generality of whether single versus multiple exposure to anesthetic agents during development has differential effects on long-term cognitive outcome. We also tested effects of single and multiple isoflurane exposure on both male and female rats.

## Materials and Methods

Experimental procedures were carried out in strict accordance with the recommendations in the Guide for the Care and Use of Laboratory Animals of the National Institutes of Health. The protocol was approved by the Institutional Animal Care and Use Committee of Mount Sinai School of Medicine (Protocol Number: LA-00071).

Postnatal day (P) 7 male or female Long–Evans rat pups, from six natural litters, were randomly allocated to one of four experimental groups, balanced primarily for dam and, as far as was possible, for sex. Rats were placed in a temperature controlled chamber and either received a single 2-h exposure to anesthesia (1A) or control condition (1C) at P7, or three 2-h exposures to anesthesia (3A) or control condition (3C), at P7, P10, and P13. Anesthesia was induced with 3% isoflurane in 100% oxygen until loss of righting reflex and response to toe and tail pinch; at which point the 2-h exposure began and consisted of 1.8% isoflurane delivered in 100% oxygen. Rats in the control condition received 100% oxygen in identical environmental conditions to rats receiving anesthesia. Monitoring included chamber oxygen, carbon dioxide, and isoflurane concentrations, and subject pulse oximetry (VitalStore, Vetronic Services Ltd., UK). Rectal temperature was monitored (PowerLab, ADInstruments Ltd., UK) and maintained at 36.5 ± 0.5°C. Rats were recovered in 100% oxygen for 20 min and returned to the dam with rats from the control condition; where they remained until weaning at 3 weeks of age. At weaning, rats were divided into same-sex groups and housed, in standard “Individually Ventilated Cages,” on a 12:12 reverse light cycle with lights off at 8 a.m.

In a separate experiment, rats that received 2 h of 100% oxygen on P7 (control group) and rats that received 2 h of 1.8% isoflurane in 100% oxygen on P7, P7 and P10 or P7, P10 and P13 were used for arterial blood gas analysis. Samples were collected at the end of the period of anesthesia (before recovery) and the pups immediately euthanized. Control rats were exposed to 3% isoflurane in 100% oxygen until loss of righting reflex (approximately 20 s) just prior to blood sampling in order to prevent distress. Rats were removed from the anesthetic chamber and a transcardial blood sample was immediately taken and analyzed (Radiometer ABL80, Cleveland, OH, USA) for pH, pCO_2_, and pO_2_. Rats from the four experimental groups (1A: five males, five females; 1C: seven males, four females; 3A: six males, five females; 3C: six males, two females) were food-restricted from P81 and maintained at 85% of age matched *ad libitum* levels. At P88 rats were acclimated to the radial arm maze (RAM) by allowing them to freely explore it for 5 min per day for 3 days and collect randomly scattered food rewards (half pieces of Cheerios cereal). A 12 arm RAM was used. Each arm of the RAM consisted of an aluminum tray, 80 cm long, 14 cm wide, and 3 cm high, with a well at the distal end containing a food reward odor mask, covered by a wire grid on top of which was placed the food reward. The wire grid was recessed to ensure that the food reward was not visible to the rat, until the arm choice had been made. At the proximal end of the arm was a manually operated clear Perspex door that led to the central chamber of the RAM. The RAM was raised 90 cm from the floor and surrounded by a curtain.

Beginning on P91 rats underwent one trial per day of RAM testing for 9 days in order to evaluate acquisition of the task. All testing occurred between 8.30 am and 7.30 pm. Spatial cues were present on the curtain for this phase. The RAM was cleaned with 25% ethanol between each rat and rotated by 30° at the end of each testing day. A win-shift procedure was used, in which each of the 12 arms was baited at the beginning of each trial, but the rewards were not replaced within each trial. The rat completed the trial when all 12 food rewards were collected or when 900 s had elapsed. Moveable doors at the entrance to each arm were used to restrict the rat to the central platform of the maze for 5 s between choices, to prevent chaining or stereotyped motor patterns. During testing, errors were scored when a rat entered an arm that it had previously entered during that trial. The following performance measures were recorded: time to complete the RAM (time required to obtain all 12 food rewards up to a maximum of 900 s); choices before the first error (number of arm choices made during a trial before the first error occurred) and omissions (number of unvisited arms at the end of a trial).

Arm entries per minute during the initial stages of testing (day 1–3) did not differ between groups, indicating that there were no differences in motor function or exploratory activity on the maze.

Data were analyzed (SPSS v19) with independent sample *t*-test (blood gases) or repeated measures ANOVA using group and sex as between subject variables and day of testing as the within subject variable (behavioral data). It was not the intention of this study to compare the effects of single versus multiple exposure to 100% oxygen on cognitive outcome; and as control group RAM data (groups 1C and 3C) were not significantly different for any parameter they were combined into a single group (group C) for comparison with the anesthesia groups. Results at the *p* = 0.05 level are considered statistically significant. In order to control for overall false discovery rate, the Benjamini and Hochberg procedure (25) was performed, to the 0.05 level, for group effects. Only group effects that are significant when the Benjamani and Hochberg procedure is performed are reported.

## Results

Blood gas values at each time point revealed moderate acidosis in each of the three anesthetic groups (P7; P7, and P10; P7, P10, and P13) compared to control rats (Table 1). Group P7 rats tended to have higher pCO_2_ (hypercapnia) than control rats (*p* = 0.09). Hypercapnia relative to control rats was detected in groups P10 and P13 and reached statistical significance (Table 1). There was no difference in pO_2_ between control and anesthetized rats. There were no differences in pH, pCO_2_, or pO_2_ between groups that received anesthesia.

**Table 1:** **Arterial blood gas parameters for control rats who received 2 h of 100% oxygen on P7; rats that received 2 h of isoflurane anesthesia on P7 (group P7), both P7 and P10 (group P10), or on P7, P10, and P13 (group P13)**.

Parameter	Group			
	P7 control (*n* = 8)	P7 (*n* = 8)	P10 (*n* = 8)	P13 (*n* = 9)
pH	7.4 ± 0.02*	7.29 ± 0.03	7.25 ± 0.05	7.26 ± 0.03
pCO_2_ (mmHg)	44.5 ± 4.1**	56.6 ± 7.8	72 ± 7.2	59.6 ± 5.8
pO_2_ (mmHg)	196.5 ± 34.2	191.8 ± 35.2	209.1 ± 50.5	212.6 ± 52.1

Values are mean ± SEM. *Rats in the three groups that received isoflurane were acidotic compared to P7 control rats (P7: p = 0.03; P10: p = 0.01; P13: p = 0.01). **Rats in group P10 and group P13 were hypercapnic compared to P7 control rats (P10: p = 0.001; P13: p = 0.05). There were no group differences in partial pressure of oxygen.

In terms of overall RAM performance, all rats took less time to complete the RAM and made fewer errors and omissions across testing days. There was a main effect of day of testing for time to complete the RAM [*F*(8,272) = 19.811, *p* < 0.0001], choices before first error [*F*(8,272) = 26.919, *p* < 0.0001], and omissions [*F*(8,272) = 21.178, *p* < 0.0001]. In other words, the performance of all rats improved across day of testing, demonstrating the absence of floor or ceiling learning effect in any group.

**Figure 1:**
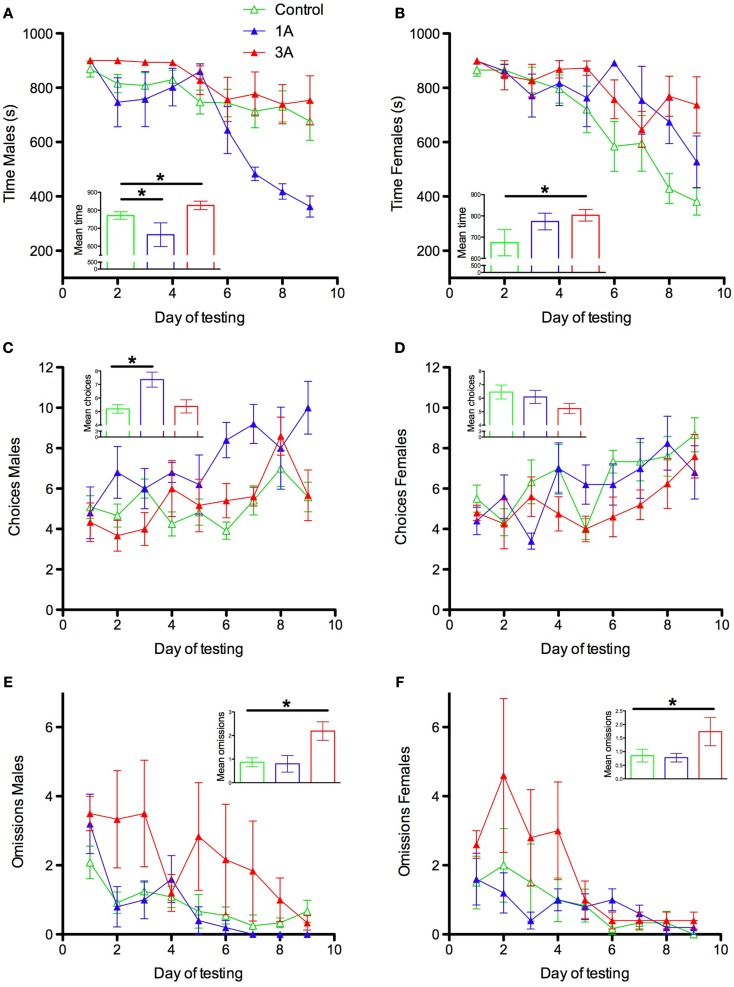
**Radial arm maze performance parameters for male (A,C,E) and female (B,D,F) rats exposed to 2 h isoflurane at postnatal day (P) 7 (group 1A) or P7, P10, and P13 (group 3A), or 2 h control condition (group C)**. Time = time required to obtain all 12 food rewards up to a maximum of 900 s; Choices = the number of arm choices made during a trial before the first error occurred; Omissions = the number of unvisited arms at the end of a trial. *p ≤ 0.05.

Different measures of maze performance revealed different patterns of anesthesia effects and unexpected sex effects. For time to complete the maze there was a main effect of group [*F*(2,34) = 4.633, *p* = 0.017] an interaction between group and sex [*F*(2,34) = 5.252, *p* = 0.01] and between day of testing, group, and sex [*F*(16,272) = 2.71, *p* = 0.001] (Figures 1A,B). These interactions were decomposed with focused ANOVAs to specifically examine the 1A and 3A groups relative to controls. Comparison of groups C and 1A revealed interactions of group and sex (*p* = 0.005) and day, group, and sex (*p* < 0.0005). This interaction represents superior performance of 1A males relative to male controls (main effect of group, *p* = 0.02, group × day interaction, *p* = 0.001) and a trend toward poorer performance of 1A females relative to female controls (group × day interaction, *p* = 0.074). 1A males outperformed 1A females (sex × day interaction for 1A rats only, *p* = 0.049). Female controls outperformed male controls (sex × day interaction for C rats only, *p* = 0.002, main effect of sex *p* = 0.033). Parallel analyses comparing groups C and 3A revealed a main effect of group (*p* = 0.014) that did not interact with sex (*p* = 0.224) or with sex and day (*p* = 0.058) so this effect was not decomposed further.

There was also a main effect of group [*F*(2,34) = 5.249, *p* = 0.01] and an interaction between sex and group [*F*(2,34) = 3.548, *p* = 0.04] for choices to first error (Figures 1C,D). Decomposition of these effects revealed a similar pattern to time, where this interaction was driven by 1A males relative to male controls. However, on this measure there was no significant difference between 3A and control (*p* = 0.098). There was a main effect of group for omissions [*F*(2,34) = 3.791, *p* = 0.033] (Figures 1E,F) that did not interact with sex or day. Group 1A did not differ significantly from group C on this measure (*p* = 0.602) but group 3A did (*p* = 0.042). These effects also did not interact with sex.

Overall, these results indicate that repeated exposure to anesthesia early in development produces persistent spatial memory impairments in both male and female rats when tested as adults. A single exposure appears to, paradoxically, improve performance of males relative to male controls on some measures, whereas once-exposed females show a trend toward impairment relative to controls, and significant impairment relative to males that were exposed once. These effects are seen against an apparent background of sex differences in control animals on these measures, with females tending to perform better than males.

## Discussion

The principal finding of this study is that repeated exposure to isoflurane during development is associated with greater long-term impairment, in the ability of adult rats to acquire and perform a spatial memory task, than single exposure. Rats of both sexes that had been repeatedly exposed were slower to complete the maze and made more errors of omission (failure to visit baited arms within the time allowed for maze completion). Single exposure to isoflurane revealed an unexpected apparent facilitatory effect in males, with single-exposed males completing the maze faster than male controls, and making more choices before the first error occurred. Collectively these findings support the view that repeated exposures to general anesthesia during development is associated with greater long-term cognitive impairment than single exposure. Furthermore, although not the focus of this study, they raise the possibility that sex may be a critical variable in the neurodevelopmental outcomes of early-life anesthetic exposure.

Population-based, retrospective birth cohort studies demonstrate that children who receive multiple (but not single) general anesthetics at a young age are at increased risk for learning disability and attention deficit disorder (18–20). This remains the case when results are adjusted for co-morbidities (19, 20). Our results are consistent with these studies, demonstrating a greater detrimental effect on cognition of multiple exposures of general anesthesia relative to a single anesthetic exposure, in the absence of surgery and other associated environmental factors that cannot be controlled for in patient studies.

Multiple exposures of neonatal rodents to isoflurane has previously been shown to cause long-term cognitive deficits. Zhu et al. (9) demonstrated that adult rats, that received 35 min of isoflurane anesthesia daily, for five consecutive days, from P14 were cognitively impaired in comparison to control animals. Our study further demonstrates that the magnitude of the deficits resulting from multiple exposures to isoflurane early in life, is greater than those resulting from a single exposure.

Anesthetic induced neuroapoptosis in the developing brain has been shown to be age-dependent, at least in rats. The greatest vulnerability in rats occurs at P7, with little or no increase in apoptosis at P10 or P14 (16). If neurocognitive impairment following anesthetic exposure is a direct result of anesthetic-induced neuroapoptosis, multiple exposure may be associated with either cumulative neuron loss, sensitization to the effects of subsequent exposures, or a combination of these factors. In particular, our results could be attributed to greater cumulative exposure in the multiple group (a total of 6 h) relative to the single group (2 h). But because the subsequent exposures occurred on days at which single exposures would not be expected to produce marked neuroapoptosis on their own, our findings suggest that a more complex mechanism is at work. Persistent dendritic spine modifications also occur following anesthetic exposure. These have been shown in rats to differ depending on the developmental stage that exposure occurs; with decreases in dendritic spine density occurring at P5 and P10 (26) and increases occurring at P15, P20, and P30 (27). On this view, the deficits in neurocognitive function in the group that received multiple exposures in our study, might be the result of apoptotic neurodegeneration on P7 combined with alterations in spine density on P10 or P13. Understanding the mechanism by which multiple anesthetic exposure, in particular, causes long-term cognitive impairment is a critical topic for future research.

Single exposure to anesthesia indicated an unexpected sex effect, with single-exposed males performing better than male controls and single-exposed females tending to perform worse than female controls, and significantly worse than single-exposed males. As sex differences were not the focus of our study, the study may not be powered adequately to detect them and it is unclear why male rats in our study who undergo a single exposure to anesthesia performed better than those who received control treatment. However, a similar effect was demonstrated by Shen et al. (22) who found that neonatal male mice exposed to a single episode of sevoflurane anesthesia had facilitated escape latency performance on the Morris water maze. Our observation is also consistent with the result of a very recent study, reporting that female but not male rats exposed to a single episode of anesthesia at P7, showed impaired acquision of place trial learning on the Morris water maze (28). Both the win-shift RAM and Morris water maze tasks test spatial memory performance. An improvement in long-term spatial reference memory has also been demonstrated in male rats that received a single exposure of isoflurane as adults (10). Sex differences in the spatial memory performance of normal (control) subjects have long been observed in many species, including humans (29) and rats (30), with the direction of the sex difference depending on the design of the behavioral task (31). In our study, control females tended to perform better than control males, and this effect reversed in single-exposed rats. A reversal of the direction of sex difference in RAM performance occurs in rats that undergo chronic stress prior to behavioral testing (32). One possibility, for the apparent improved performance of once-exposed males in our study, is therefore that the control condition (2 h of maternal separation) in our experiment provided a stressor. The resulting reversal of the sex effect in control animals would therefore lead to an apparent but false improvement in performance in once-exposed males relative to controls. This explanation seems unlikely, given that the stress paradigms used to demonstrate this effect involve a chronic stressor administered over days rather than hours. Future experiments could, however, be designed to exclude this possibility completely by only briefly separating control animals from the dam.

Sex differences in rodent post-anesthetic cognitive impairment have been demonstrated. Mice exposed to isoflurane anesthesia at P0 exhibit a sex difference, with the performance of female mice being less affected than males, when tested on a RAM task at P58, an earlier time point to that of our study (8). As mentioned previously, potential mechanisms for post-anesthetic cognitive impairment in neonates include anesthetic induced alterations in neuronal ultrastructure. To date, such effects of neonatal exposure to anesthesia have only been studied in males but sex is known to interact with other physiological stressors that lead to alterations in rodent neuronal ultrastructure. Hippocampal CA1 dendritic spine density in male versus female rats responds in the opposite direction to the same physiological stimulus (33) and alterations of prefrontal cortex neuronal structure in the offspring of dams that undergo gestational stress, occur in a sex-specific manner (34). We propose that sex may interact with the effect of anesthesia on dendritic spine morphology (as do other physiological stressors) resulting in sex-dependent effects on long-term cognitive outcome. This provides a potential mechanism by which sex could affect the developmental time course of post-anesthesia cognitive impairment. Further studies specifically designed to investigate behavioral sex differences are required to fully characterize and ascertain the importance of these observations.

There are a number of important considerations when interpreting the results of our study. The number of rats of each sex is low, it is therefore possible that we were underpowered to detect sex differences. It is of note, however, that the facilitatory effect seen in single-exposed males has been previously demonstrated (22) and that performance of female rats trended in the opposite direction, which we think indicates that these results warrant further investigation.

We chose to expose rat pups from postnatal day 7. This developmental time point is commonly used to investigate neonatal anesthetic neurotoxicity; in part because the peak of synaptogenesis occurs around this age in rats (35) and is thought to be related to the period of greatest vulnerability. The correspondence of this age to a specific neurodevelopmental stage in humans depends on which measures are used to make the comparison. In a recent review article, the inherent difficulties with making such comparisons are discussed. According to morphologic measures, postnatal day 2–7 in rats corresponds to the human third trimester, and the brain growth spurt occurring at birth in humans is centered around P7 in rats (36). However, according to their own neuroinformatics model (37), which integrates a number of different measures into a computational model, the stage of development of the brain in humans at birth is equivalent to P10 in rats. It may be, therefore, that our study models multiple anesthetic exposures beginning at a stage (late third trimester), that anesthesia is rarely needed. The stage of pregnancy, however, for parturition of surviving premature human infants is decreasing as advances in critical care progress. Infants born as early as the middle of the second trimester may now survive (38, 39). Many of these infants receive sedation and anesthesia as part of their care. We therefore take the view, that the choice of P7 as a stage of anesthetic exposure in rats, represents an increasingly clinically relevant experimental model, regardless of the precise correspondence to stages of human brain development.

Hypercapnia and acidosis were observed in anesthetized rats at each time point in our study. Whilst this demonstrates anesthetic induced physiological impairment, it is highly unlikely that this could have resulted in the behavioral differences demonstrated in our study. Equivalent levels of hypercapnia and acidosis were seen in rats that received a single exposure to anesthesia and multiple exposures to anesthesia. For this reason we conclude that the spatial memory deficits seen in the multiple anesthetic exposure group as compared to the single exposure group are not due to respiratory disturbances. Additionally, it has been demonstrated that rats exposed to 4 h of carbon dioxide at P7 do not develop long-term spatial memory deficits (40), showing that hypercapnia alone is not a sufficient causal factor.

Rats in our study demonstrated a venous partial pressure of oxygen of approximately 200 mmHg. Rats were exposed to 100% oxygen as the carrier gas and would therefore be expected to demonstrate venous partial pressures of oxygen approximately three times higher. The lower values however, reflect the fact that rat pups were removed from the anesthetic chamber for blood gas sampling, and are not therefore indicative of pulmonary disease. Delivery of anesthetic via a mask would eliminate this potential confound for future studies.

In order to avoid the potential confound of gonadectomy (41), we used intact female rats and so any sex effect is potentially confounded by hormonal variation. We also chose to test all subjects at exactly the same developmental time point rather than at a particular stage of estrus. However, it is of note that the increase in variability that these factors introduce, would not increase the likelihood that an effect would be detected between females in the three anesthetic groups.

The immediate implication of our results is that a single short (2-h) exposure to isoflurane produces little or no long-term cognitive impairment relative to multiple short exposures; and that multiple short exposures are sufficient to cause long-lasting (3 months post exposure) cognitive impairment in rats. Furthermore, the long-term cognitive effects of early anesthetic exposure may differ between the sexes, suggesting sex as a critical variable for investigation in future studies of developmental anesthetic neurotoxicity, to confirm and extend or refute these results.

## Conflict of Interest Statement

The authors declare that the research was conducted in the absence of any commercial or financial relationships that could be construed as a potential conflict of interest.
